# Leaf Morphology and Ultrastructure Responses to Elevated O_3_ in Transgenic Bt (cry1Ab/cry1Ac) Rice and Conventional Rice under Fully Open-Air Field Conditions

**DOI:** 10.1371/journal.pone.0082199

**Published:** 2013-12-06

**Authors:** Chunyan Li, Biao Liu, Chunhua Li, Qing Zeng, Mingzhuo Hao, Zhengmin Han, Jianguo Zhu, Xiaogang Li, Wenjing Shen

**Affiliations:** 1 College of Forest Resources and Environment, Nanjing Forestry University, Nanjing, Jiangsu, China; 2 Key Biosafety Laboratory in Nanjing Institute of Environmental Sciences, Ministry of Environmental Protection of China, Nanjing, Jiangsu, China; 3 State Key Laboratory of Soil and Sustainable Agriculture, Institute of Soil Sciences, Chinese Academy of Sciences, Nanjing, Jiangsu, China; 4 Liaoning Agricultural College, Yingkou, Liaoning, China; 5 Key Laboratory of Soil Environment and Pollution Remediation, Institute of Soil Sciences, Chinese Academy of Sciences, Nanjing, Jiangsu, China; University of Nottingham, United Kingdom

## Abstract

**Background:**

Elevated tropospheric ozone severely affects not only yield but also the morphology, structure and physiological functions of plants. Because of concerns regarding the potential environmental risk of transgenic crops, it is important to monitor changes in transgenic insect-resistant rice under the projected high tropospheric ozone before its commercial release.

**Methodology/Principal Findings:**

Using a free-air concentration enrichment (FACE) system, we investigated the changes in leaf morphology and leaf ultrastructure of two rice varieties grown in plastic pots, transgenic Bt Shanyou 63 (Bt-SY63, carrying a fusion gene of cry1Ab and cry1Ac) and its non-transgenic counterpart (SY63), in elevated O_3_ (E-O_3_) versus ambient O_3_ (A-O_3_) after 64-DAS (Days after seeding), 85-DAS and 102-DAS. Our results indicated that E-O_3_ had no significant effects on leaf length, leaf width, leaf area, stomatal length and stomatal density for both Bt-SY63 and SY63. E-O_3_ increased the leaf thickness of Bt-SY63, but decreased that of SY63. O_3_ stress caused early swelling of the thylakoids of chloroplasts, a significant increase in the proportion of total plastoglobule area in the entire cell area (PCAP) and a significant decrease in the proportion of total starch grain area in the entire cell area (SCAP), suggesting that E-O_3_ accelerated the leaf senescence of the two rice genotypes. Compared with SY63, E-O_3_ caused early swelling of the thylakoids of chloroplasts and more substantial breakdown of chloroplasts in Bt-SY63.

**Conclusions/Significance:**

Our results suggest that the incorporation of cry1Ab/Ac into SY63 could induce unintentional changes in some parts of plant morphology and that O_3_ stress results in greater leaf damage to Bt-SY63 than to SY63, with the former coupled with higher O_3_ sensitivity in CCAP (the proportions of total chloroplast area in the entire cell area), PCAP and SCAP. This study provides valuable baseline information for the prospective commercial release of transgenic crops under the projected future climate.

## Introduction

Tropospheric ozone (O_3_) causes severe damage to crop production and is recognized as the most phytotoxic air pollutant in many areas of the world[1-4]. The tropospheric O_3_ concentration ([O_3_]) has now reached a global mean of approximately 50 nl•l^-1^ (ppb), which exceeds 40 ppb, the threshold at which vegetation that are sensitive to O_3_ pollution are affected [5,6]. The value of tropospheric [O_3_], moreover, is predicted to continue to increase by 20-25% between 2015 and 2050 and to increase by 40-60% by 2100 [7]. Rice (*Oryza sativa* L.) is the most important food crop in the world [8]. Like other staple crop species, rice is sensitive to O_3_ pollution [9,10]. Feng et al. [10] reported that the yield loss in rice would be projected to be 27% at an [O_3_] of 51-75 ppb.

Researchers have also found that elevated O_3_ severely affects not only yield but also the morphology, structure, physiological functions and reproduction of plants [11-13]. Furthermore, stomata play an important role in damage caused by O_3_ because O_3_ uptake in plants is strongly mediated by stomata. Many studies suggest that certain leaf morphological characteristics such as stomatal density and leaf thickness may be related to differences in O_3_ sensitivity [14,15]. In addition, many studies of O_3_’s effects on *Glycine max* L., *Raphanus sativus* L., and *Spinacia oleracea* L. showed that the prominent alterations caused by O_3_ exposure were thylakoid swelling and an increase in plastoglobuli after O_3_ entered into leaves through stomata [16–18]. To our knowledge, the effects of elevated O_3_ on leaf morphology and ultrastructure in rice are unknown.

Since security certificates for two varieties of transgenic insect-resistant rice, Huahui1 and Bt Shanyou 63 (Bt-SY63), were officially awarded in China in 2009, concerns regarding the potential ecological and environmental risk of transgenic rice have been raised. Our recent trial confirmed that photosynthetic parameters changed to a greater extent in the rice leaves of Bt-SY63 compared with its non-transgenic counterpart Shanyou 63 (SY63) under O_3_-FACE (O_3_-Free Air Concentration Enrichment) conditions [19]. Moreover, the results of this trial suggested that Bt-SY63 is more sensitive to elevated [O_3_] than SY63, with the former variety coupled with great volatility [19]. The objective of this study was to investigate changes in the leaf surface morphology and internal ultrastructure of Bt-SY63 and SY63 under O_3_ free air-controlled enrichment conditions in order to determine the different responses generated by transgenic rice and its non-transgenic traditional counterpart when they are exposed to elevated tropospheric O_3_.

## Materials and Methods

### Experimental site and O_3_-FACE system

The experiment was conducted at O_3_-FACE facilities, located in the town of Xiaoji, Jiangdu county, Jiangsu province, China (119°42′0″E, 32°35′5″N), in a continuous rotation ecosystem with rice and wheat from June 2010 to September 2010. The site is in a subtropical marine climate with a mean annual precipitation of 980 mm, mean annual evaporation >1100 mm, annual mean air temperature of 14.9°C, total annual sunshine time >2100 h, and a frostless period >220 days. 

**Figure 1:**
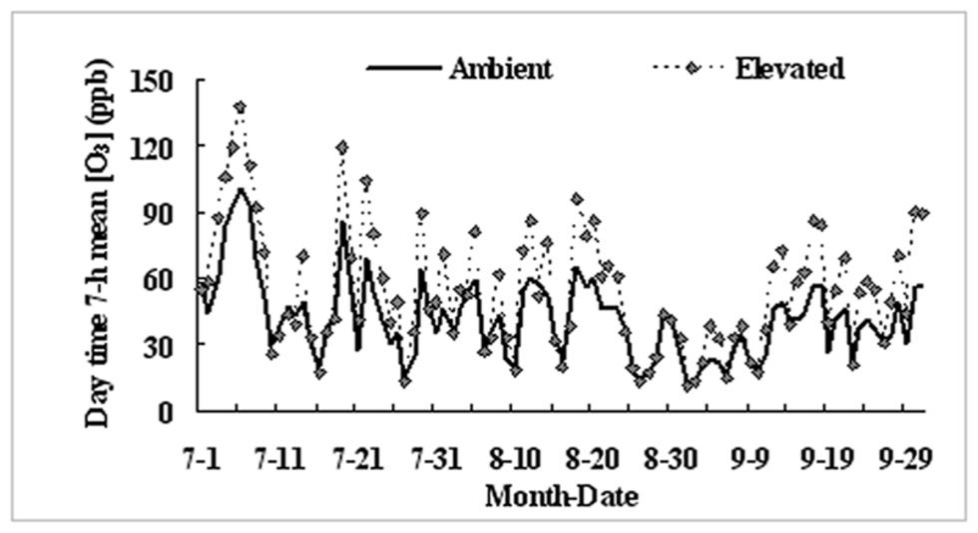
Changes in [O_3_] in ambient and elevated plots in 2010.

The O_3_-FACE system has six plots, of which three were under ambient [O_3_] (A-O_3_) and three were under elevated [O_3_] (E-O_3_). Each E-O_3_ plot was enclosed with a 14 m diameter octagonal ring that consisted of eight 6-m-long ABS horizontal pipes. In the E-O_3_ plots, O_3_ gas was injected into the air via tiny holes (0.3 mm in diameter) in the ABS pipes at about 0.5 m above the canopy from 9:00 a.m. to sunset in the sunshine, and the target [O_3_] was maintained at 50% higher than the ambient [O_3_], controlled by a computer. Each of the E-O_3_ plots was separated from the other plots by at least 70 m to avoid cross-contamination. In the A-O_3_ plots, plants were grown under ambient [O_3_] without the ring structures. For more details of the O_3_ fumigation system, see 20. Fumigation began on 2 July 2010 in the E-O_3_ plots in this study. The changes in [O_3_] in the elevated and ambient plots during the entire experimental period in 2010 are shown in Figure 1. The experiment was permitted by the Biosafety Committee of Nanjing Institute of Environmental Sciences, Ministry of Environmental Protection of China as well as the Institute of Soil Sciences, Chinese Academy of Sciences.

### Plant materials and crop cultivation

The two rice varieties, Bt-SY63 and SY63, used in this experiment were kindly provided by Huazhong Agricultural University. Bt-SY63 possesses a fusion gene derived from Cry1Ab and Cry1Ac, which is primarily used to control lepidopteron pests such as rice-stem borer. Rice seeds were sown on 13 June 2010. The seedlings were manually transplanted into plastic pots (25 cm tall × 23 cm long × 18 cm wide) on 23 June, two plants per pot, and then were placed randomly in E-O_3_ and A-O_3_ plots. The plastic pots were in a row (equivalent to 24 pots•m^-2^) and semi-buried, and the water level was maintained 2–3 cm above the soil surface until the end of the experiment. During the pollen stage of the Bt-SY63, from 8 August to 20 August, the rice spikes were covered by translucent paper bags to prevent the pollens from dispersing.

### Morphology measurement

The leaf area meter (AM100, ADC, UK) was adopted to measure the leaf length (cm), leaf width (cm) and leaf area (cm^2^) values from the second upper fully expanded functional leaf of each variety in each plot (equivalent to each treatment) on 64-DAS (16 August, 44 d of O_3_ treatment), 85-DAS (6 September, 65 d of O_3_ treatment) and 102-DAS (23 September, 82 d of O_3_ treatment), respectively. The total values of 10 leaves from 10 plants per treatment were averaged as the mean leaf length, width and area. The leaf thickness was measured according to the method described in [21]. 

The stomatal length (µm) and stomatal density (number•cm^−2^) from the second functional leaf were measured as follows: patches of about 2 cm^2^ around the midpoint of the leaf blade were removed from the second upper functional leaves, adhered on the specimen surface, and then observed and photographed under a “HITACHI Table Top Scanning Microscope TM-1000”. Stomatal density and stomatal length were calculated from five fully expanded leaves per treatment. On each leaf, five random areas were examined at ×400 magnification for stomatal density, and three random areas were examined at ×1000 magnification for stomatal length.

### Transmission electron microscopy

The same leaves used to determine stomatal densities and stomatal length were used for electron microscopy. The middle part of the leaves away from the midrib was cut into small pieces (about 1.5 mm^2^). These fragments were fixed in a bottle with 5% glutaraldehyde in 0.1 M sodium phosphate buffer, pH 7.0. After 4 h at 0-4°C and three rinses with buffer, the samples were post-fixed in 2% OsO_4_ and then rinsed again with the same buffer for the same amount of time. Thereafter, the samples were dehydrated in an acetone series (30%, 50%, 70%, 90%; 30 min each) and 100% acetone (two times, 30 min each) and embedded in Epon 812 resin. The samples were cut into thin sections of approximately 50-70 µm with a LKB-V ultramicrotome (Sweden), and thin sections were stained on copper grids with lead citrate and uranyl acetate before being examined by a Hitachi 600-A-2 (Japan) transmission electron microscope operated at 75 kV. 

Ten representative mesophyll cell sections at ×5000 magnification (2 µm) from each of the three replicate plants per sampling time per treatment were analyzed for cell wall thickness, sectional areas of mesophyll cells, chloroplasts, starch grains and plastoglobuli with Image-Pro Plus 6.0 (Media Cybernetics, MD, USA). With respect to the sectioning of three-dimensional organelles, the proportions of total chloroplast area in the entire cell area (%) (CCAP), total plastoglobule area in the entire cell area (%) (PCAP), and total starch grain area (%) (SCAP) in the entire cell area were calculated as the final result in order to minimize measurement errors.

### Statistical analyses

Data were analyzed using SPSS software (SPSS Inc., version 16.0). Data were first normalized and then checked for normal distributions (Kolmogorov–Smirnov test) and homogeneity of variance (Levene's test). The normalizing transformation succeeded to improve the non-normality in the data. The repeated measures procedure of the general linear model (making the concentration of O_3_ and rice variety as independent factors, growth stage as repeated measures factor), a two-way ANOVA with repeated measures, was applied in the following characteristics of rice: leaf length, leaf width, leaf area, leaf thickness and mesophyll cell wall thickness. The repeated measures procedure of the general linear model failed to be employed in the analysis of the three parameters CCAP, PCAP and SCAP owing to severely degenerated chloroplasts at 102-DAS, which led to an inability to calculate the three abovementioned parameters. As a result, a two-way ANOVA without repeated measures was used to determine the effects of O_3_, variety and their interactions on CCAP, PCAP and SCAP in every separate growth stage.

## Results

### Leaf length, leaf width and leaf area

The repeated measures procedure of the general linear model showed that significant differences were observed in leaf length (*P* < 0.05) and leaf area (*P* < 0.05) between Bt-SY63 and SY63, while no significant difference in leaf width (*P* > 0.05) was found between the two varieties (Table 1). The leaf length and leaf area of Bt-SY63 were significantly (*P* < 0.05) smaller than those of SY63. Time (growth stage) showed a significant effect on leaf length (*P* < 0.05), that is leaf length reduced significantly along with leaf senescence. No significant time effects (*P* > 0.05) were found on leaf width and leaf area. E-O_3_ had no significant effects (*P* > 0.05) on the leaf length, leaf width and leaf area of the two rice genotypes. The effects of the interaction between variety and O_3_, the interaction between time and O_3_, the interaction between time and variety, and the combined effect of the three factors were all non-significant (*P* > 0.05) on the leaf length, leaf width and leaf area of the two rice genotypes. 

**Table 1:** Changes in leaf length, leaf width and leaf area (Means ± S.D.) in Bt-SY63 and SY63 under ambient and elevated O_3_.

		**Leaf length (cm)**			**Leaf width (cm)**			**Leaf area (cm^2^)**		
**Variety**	**Treatment**	**64-DAS**	**85-DAS**	**105-DAS**	**64-DAS**	**85-DAS**	**105-DAS**	**64-DAS**	**85-DAS**	**105-DAS**
Bt-SY63	A-O_3_	48.27±1.38	47.43±2.29	46.95±2.71	1.62±0.29	1.84±0.09	1.84±0.03	58.68±11.54	65.88±5.76	64.68±3.25
	E-O_3_	52.11±3.61	46.60±1.72	49.37±3.15	1.84±0.15	1.89±0.13	1.87±0.08	72.52±9.98	66.16± 5.76	69.48±7.35
SY63	A-O_3_	55.23±4.24	54.89±1.21	46.47±4.09	1.89±0.08	1.96±0.04	1.81±0.08	77.96± 8.88	80.63±3.30	67.54±6.60
	E-O_3_	54.17±4.02	51.19±6.87	49.07±3.40	1.84±0.14	1.88±0.11	1.83±0.10	74.56±0.25	72.50±13.16	63.36±8.17
**Effect**		**Significance of repeated measures of general linear model**								
		**Leaf length**			**Leaf width**			**Leaf area**		
O_3_		n.s.			n.s.			n.s.		
Variety		*			n.s.			*.		
O_3_ × variety		n.s.			n.s.			n.s.		
Time		*			n.s.			n.s.		
Time × O_3_		n.s.			n.s.			n.s.		
Time × variety		n.s.			n.s.			n.s.		
Time × O_3 ×_ variety		n.s.			n.s.			n.s.		

A-O_3_, Ambient O_3_; E-O_3_, Elevated O_3_. Mean values and standard deviation of ten replicates are presented.

ANOVA: * *p* < 0.05, n.s. = not significant.

### Stomatal length and stomatal density

Similar to the observation of ANOVA results in leaf width, differences (*P* > 0.05) were observed for the stomatal length and stomatal density of both Bt-SY63 and SY63 (Table 2). 

**Table 2:** Changes in stomatal length and stomatal density (Means ± S.D.) in Bt-SY63 and SY63 under ambient and elevated O_3_.

		**Stomatal length (μm )**			**Stomatal density (number•cm^-2^)**		
**Variety**	**Treatment**	**64-DAS**	**85-DAS**	**105-DAS**	**64-DAS**	**85-DAS**	**105-DAS**
Bt-SY63	A-O_3_	21.3±2.0	22.0±1.6	22.7±1.1	4.7±0.3	4.5±0.3	3.6±0.6
	E-O_3_	22.7±1.3	23.5±1.2	23.2±1.8	4.1±0.3	4.3±0.2	3.6±0.8
SY63	A-O_3_	24.9±1.2	23.9±1.8	22.8±1.3	4.5±0.6	4.0±0.2	4.0±0.8
	E-O_3_	23.9±1.4	23.7±1.5	22.7±2.6	4.3±0.7	4.2±0.4	4.0±0.7
**Effect**		**Significance of repeated measures of general linear model**					
		**Stomatal length**			**Stomatal density**		
O_3_		n.s.			n.s.		
Variety		n.s.			n.s.		
O_3_ × variety		n.s.			n.s.		
Time		n.s.			n.s.		
Time × O_3_		n.s.			n.s.		
Time × variety		n.s.			n.s.		
Time × O_3 ×_ variety		n.s.			n.s.		

A-O_3_, Ambient O_3_; E-O_3_, Elevated O_3_. Mean values and standard deviation of ten replicates are presented.

ANOVA: * *p* < 0.05, ** p < 0.01, n.s. = not significant

### Leaf thickness

As shown in Table 3, neither O_3_ nor variety showed significant influence (*P* > 0.05) on leaf thickness. No significant effects (*P* > 0.05) of time, the interaction between time and O_3_, the interaction between time and variety, and the combined effect of the three factors were found on the leaf thickness of both Bt-SY63 and SY63. Variety and O_3_ showed significant interaction on leaf thickness (*P* < 0.05), which indicated that leaf thickness response to ozone was different for the two rice genotypes. That is, E-O_3_ increased the leaf thickness of Bt-SY63 by 4.5% relative to A-O_3_, whereas decreased that of SY63 by 4%.

**Table 3:** Changes in leaf thickness and mesophyll cell wall thickness (Means ± S.D.) in Bt-SY63 and SY63 under ambient and elevated O_3_.

		**Leaf thickness (μm )**			**Mesophyll cell wall thickness (um)**		
**Variety**	**Treatment**	**64-DAS**	**85-DAS**	**105-DAS**	**64-DAS**	**85-DAS**	**105-DAS**
Bt-SY63	A-O_3_	273.01±8.16	283.76±11.37	299.08±15.71	0.19±0.01	0.24±0.02	0.22±0.03
	E-O_3_	301.97±8.31	295.91±2.03	296.55±22.67	0.26±0.05	0.22±0.05	0.20±0.01
SY63	A-O_3_	303.97±16.26	304.08±21.26	296.40±12.48	0.25±0.02	0.22±0.03	0.24±0.02
	E-O_3_	292.57±6.72	286.59±1.75	289.13±10.14	0.23±0.02	0.25±0.01	0.23±0.02
**Effect**		**Significance of repeated measures of general linear model**					
		**Leaf thickness**			**Mesophyll cell wall thickness**		
O_3_		n.s.			n.s.		
Variety		n.s.			*		
O_3_ × variety		*			n.s.		
Time		n.s.			n.s.		
Time × O_3_		n.s.			n.s.		
Time × variety		n.s.			n.s.		
Time × O_3 ×_ variety		n.s.			*		

A-O_3_, Ambient O_3_; E-O_3_, Elevated O_3_. Mean values and standard deviation of ten replicates are presented.

ANOVA: * *p* < 0.05, ** p < 0.01, n.s. = not significant.

### Changes in leaf ultrastructure

The repeated measures procedure of the general linear model confirmed that the cell wall thickness of the mesophyll of Bt-SY63 was significantly (*P* < 0.05) smaller than that of SY63 (Table 3). The cell wall thickness of both Bt-SY63 and SY63 was not significantly affected (*P* > 0.05) by E-O_3_. Although the effects of time, the interaction between variety and O_3_, the interaction between time and O_3_, and the interaction between time and variety were all non-significant (*P* > 0.05), the combined effect of variety, O_3_ and time was significant (*P* < 0.05) on cell wall thickness. 

As shown in Table 4, at 64-DAS, E-O_3_ resulted in a significant increase in the proportion of total plastoglobule area in the entire cell area (%) (PCAP) (*P* < 0.01) and a significant decrease in the proportion of total starch grain area in the entire cell area (%) (SCAP) (*P* < 0.05), while no significant O_3_ effect (*P* > 0.05) was detected in the proportion of total chloroplast area in the entire cell area (%)(CCAP) of both Bt-SY63 and SY63. No significant variety effects (*P* > 0.05) were found in PCAP and SCAP, whereas varieties differed significantly (*P* < 0.05) in CCAP. The effects of the interaction between O_3_ and variety were all non-significant (*P* > 0.05) in CCAP, PCAP and SCAP. 

**Table 4:** Ultrastructural changes (Means ± S.D.) in Bt-SY63 and SY63 under ambient and elevated O_3_ (CCAP: the proportions of total chloroplast area in the entire cell area (%); PCAP: the proportion of total plastoglobule area in the entire cell area (%); SCAP: the proportion of total starch grain area in the entire cell area (%)).

		**CCAP (%)**			**PCAP (%)**			**SCAP (%)**		
**Variety**	**Treatment**	**64-DAS**	**85-DAS**	**102-DAS**	**64-DAS**	**85-DAS**	**102-DAS**	**64-DAS**	**85-DAS**	**102-DAS**
Bt-SY63	A-O_3_	47.53±7.47	36.10±4.56	None	0.45±0.05	1.45±0.54b	1.17±0.70	3.53±1.23	0.93±0.18	0.36±0.32
	F-O_3_	63.42±10.82	77.44±8.92	None	1.03±0.19	4.03±2.13a	none	1.80±1.10	0.41±0.25	none
SY63	A-O_3_	76.18±2.68	46.01±7.80	25.76±0.15	0.57±0.29	0.38±0.05b	0.27±0.20	5.85±3.20	1.59±0.29	0.64±0.03
	F-O_3_	72.28±12.31	56.79±7.69	32.91±11.8	1.62±0.25	2.00±0.32ab	1.02±0.55	2.32±1.60	1.09±0.06	1.09±0.13
		**Significance of two-way ANOVA**								
O_3_		n.s.	**	none	**	*	none	*	**	none
Variety		*	n.s.	none	n.s.	*	none	n.s.	**	none
O_3_ × variety		n.s.	**	none	n.s.	*	none	n.s.	*	none

A-O_3_, Ambient O_3_; E-O_3_, Elevated O_3_. Mean values and standard deviation of ten replicates are presented.

Two-way ANOVA: * *p* < 0.05, ** *p* < 0.01, n.s. = not significant.

None means an inability to determine the effect of the parameter owing to severely degenerated chloroplasts at this growth stage

**Figure 2:**
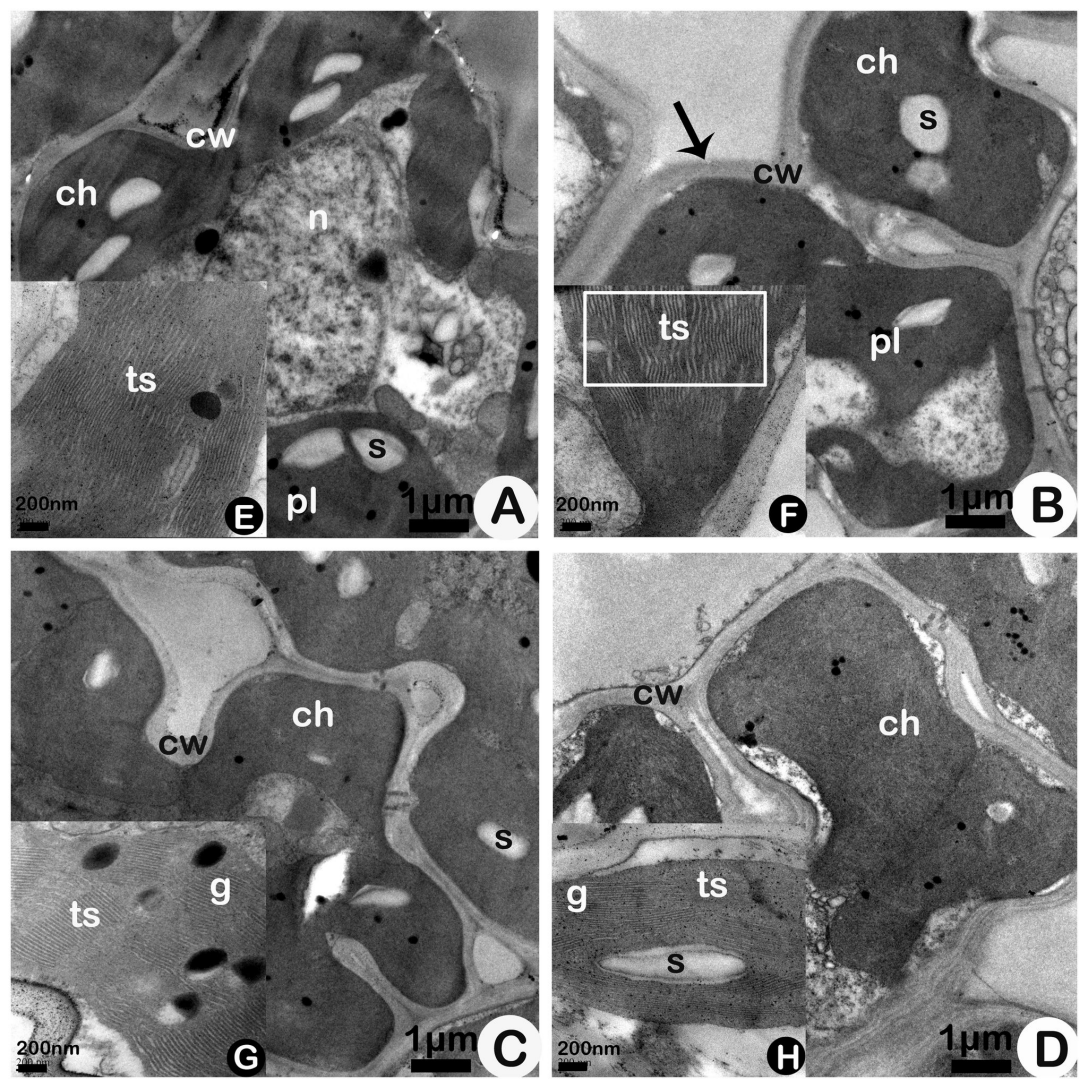
Ultrastructures of mesophyll cells (A, B, C, D) and chloroplasts (E, F, G, H) of transgenic rice Bt Shanyou 63 (Bt-SY63) (A, B) and its non-transgenic counterpart Shanyou 63 (SY63) (C, D) at 64-DAS (Days after seeding) in elevated O_3_ (B, D) and ambient O_3_ (A, C) conditions. The black arrowhead (in B) indicates the thickened mesophyll cell wall of Bt-SY63 under elevated O_3_. The white-bordered rectangle indicates a swollen thylakoid in Bt-SY63 under elevated O_3_. Abbreviations: ch, chloroplast; cw, cell wall; pl, plastoglobuli; s, starch deposits; ts, thylakoid structures; g, grana; n, nucleolus. Bar: A, B, C, D - 1 μm; E, F, G, H - 200 nm.

In addition, at 64-DAS, E-O_3_ resulted in swollen thylakoids (Figure 2F), whole fuzzy chloroplasts, and rough mesophyll cell wall (shown as an arrowhead) in Bt-SY63 (Figure 2B) compared with those observed under A-O_3_ (Figure 2A and 2E). However, in SY63, the chloroplasts were still clear under E-O_3_ (Figure 2D and 2H) compared with those observed under A-O_3_ (Figure 2C and 2G). In addition, Bt-SY63 under E-O_3_ had fuzzier whole chloroplasts than SY63 under E-O_3_. 

At 85-DAS, as shown in Table 4, no significant variety effect (*P* > 0.05) was detected in CCAP, while significant variety effects were found in PCAP (*P* < 0.05) and SCAP (*P* < 0.01). E-O_3_ resulted in significant increases in CCAP (*P* < 0.01) and PCAP (*P* < 0.05), and a significant decrease in SCAP (*P* < 0.01) of both Bt-SY63 and SY63. The effects of the interaction between O_3_ and variety were all significant in CCAP (*P* < 0.01), PCAP (*P* < 0.05) and SCAP (*P* < 0.05). This result indicates that the sensitivity of the three abovementioned parameters to O_3_ is significantly different between the two rice genotypes. E-O_3_ increased CCAP in Bt-SY63 and SY63 by 114.5% and 23.4%, respectively, comparing to A-O_3_. The corresponding increases in PCAP were 177.9% and 44.9%, respectively. E-O_3_ decreased SCAP in Bt-SY63 and SY63 by 55.9% and 31.4%, respectively, comparing to A-O_3_. 

**Figure 3:**
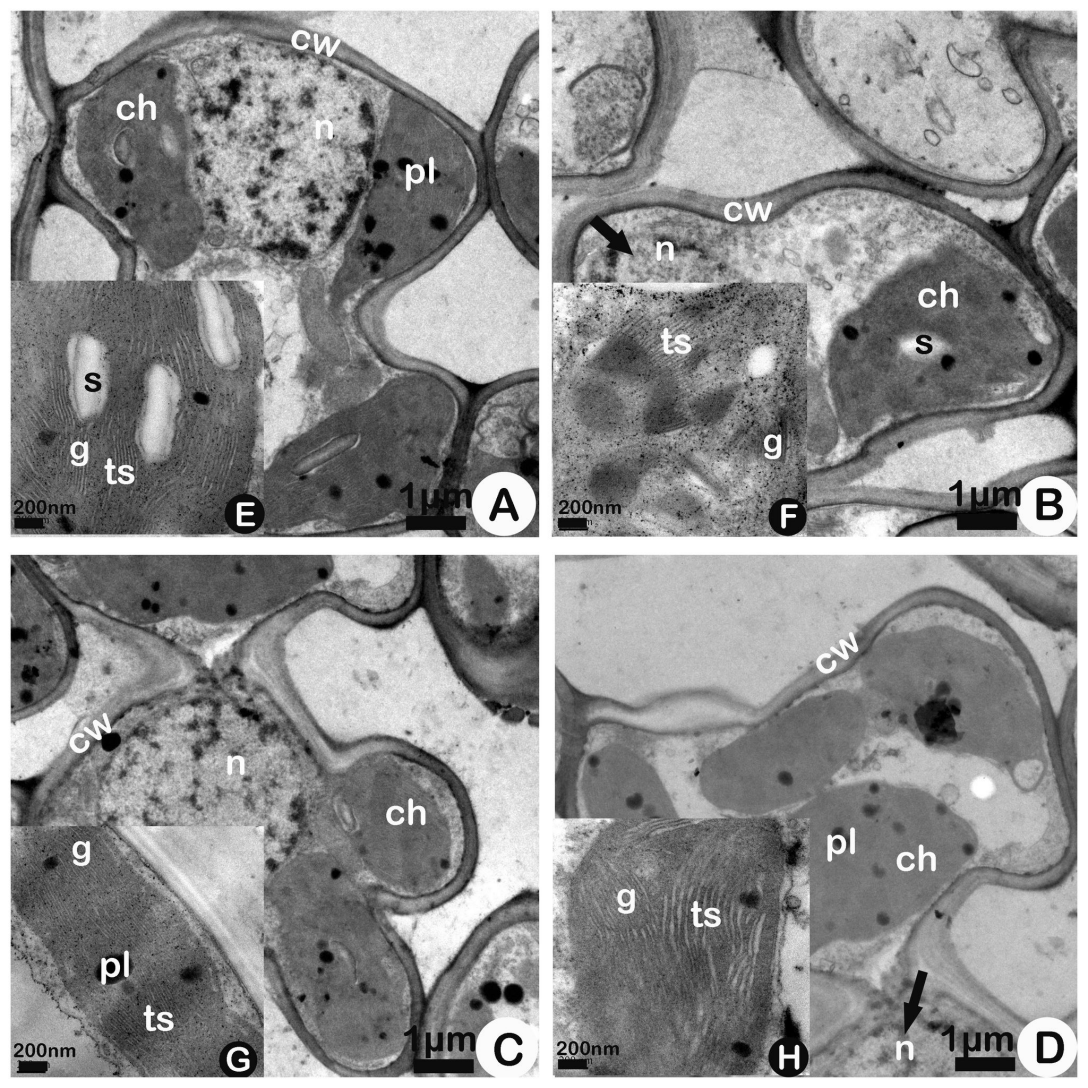
Ultrastructures of mesophyll cells (A, B, C, D) and chloroplasts (E, F, G, H) of transgenic rice Bt Shanyou 63 (Bt-SY63) (A, B) and its non-transgenic counterpart Shanyou 63 (SY63)(C, D) at 85-DAS (Days after seeding) in elevated O_3_ (B, D) and ambient O_3_ (A, C) conditions. The black arrowhead indicates the injured nuclear membranes of Bt-SY63 (in B) and SY63 (in D) under elevated O_3_. Abbreviations: ch, chloroplast; cw, cell wall; pl, plastoglobuli; s, starch deposits; ts, thylakoid structures; g, grana; n, nucleolus. Bar: A, B, C, D - 1 μm; E, F, G, H - 200 nm.

As shown in the ultrastructural images on 85-DAS in Figure 3, compared to those on 64-DAS, Bt-SY63 (Figure 3A) and SY63 (Figure 3C) under A-O_3_ did not exhibit any abnormalities at the ultrastructural level of their cellular structure except for smaller electronic density; That is, Bt-SY63 and SY63 under A-O_3_ still showed an uninjured chloroplast envelope and nuclear membrane and other distinct organelles. E-O_3_ produced injured nuclear membranes in Bt-SY63 (Figure 3B, black arrowhead) and SY63 (Figure 3D, black arrowhead) and swollen thylakoids (Figure 3H) and whole fuzzy chloroplasts (Figure 3D) in SY63. In addition, the chloroplasts in Bt-SY63 (Figure 3B) were fuzzier than those in SY63 (Figure 3D) under E-O_3_. However, grana in chloroplasts in Bt-SY63 and SY63 were still identified under both air conditions (Figure 3E, Figure 3F, Figure 3G, Figure 3H).

**Figure 4:**
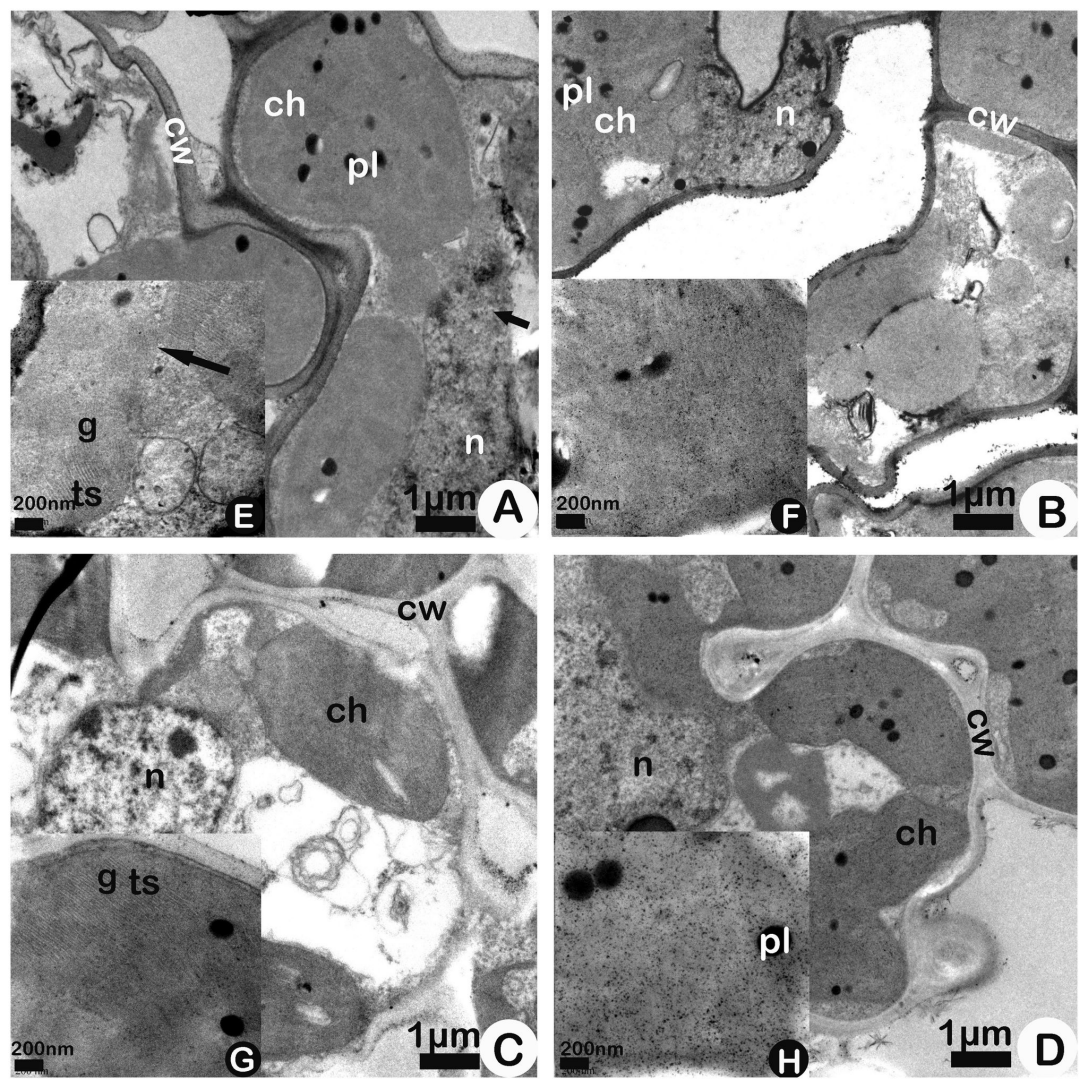
Ultrastructures of mesophyll cells (A, B, C, D) and chloroplasts (E, F,G, H) of transgenic rice Bt Shanyou 63 (Bt-SY63) (A, B) and its non-transgenic counterpart Shanyou 63 (SY63)(C, D) at 102-DAS (Days after seeding) in elevated O_3_ (B, D) and ambient O_3_ (A, C) conditions. Abbreviations: ch, chloroplast; cw, cell wall; pl, plastoglobuli; s, starch deposits; ts, thylakoid structures; g, grana; n, nucleolus. Bar: A, B, C, D - 1 μm; E, F, G, H - 200 nm.

The 102-DAS sampling time was close to harvesting time. Compared to those at 85-DAS, for Bt-SY63 and SY63 under A-O_3_ at this stage, cells and chloroplasts began to degenerate differently, thylakoids became swollen (Figure 4E, Figure 4G), the nuclear membrane became damaged in Bt-SY63 (Figure 4A, short arrow), and chloroplast envelope injury was present (Figure 4E, long arrow) in Bt-SY63. The CCAP of Bt-SY63 could not be measured because of the injured chloroplast envelope under A-O_3_, while SY63 (Figure 4C) showed uninjured chloroplast envelope under A-O_3_. However, the most prominent alterations caused by E-O_3_ in Bt-SY63 (Figure 4B) were severely degenerated chloroplasts, which occupied the entire cell and presented a patchy distribution. Under E-O_3_, the abnormal proliferation of swollen thylakoids resulted in severely degenerated chloroplasts in Bt-SY63. Moreover, these thylakoids were not organized in grana, as observed for Bt-SY63 under E-O_3_ (Figure 4F), and were often accompanied by increased stroma granulation (which led to an inability to calculate the three abovementioned parameters). Some of these alterations in Bt-SY63, such as thylakoid swelling, stroma granulation, and unidentified grana, also appeared in SY63 (Figure 4D and Figure 4H) under E-O_3_, but the chloroplasts of SY63 under E-O_3_ still exhibited a certain shape. A two-way ANOVA without repeated measures failed to be employed in the analysis of the three parameters CCAP, PCAP and SCAP owing to severely degenerated chloroplasts at this stage, which led to an inability to calculate the three abovementioned parameters.

## Discussion

Although many authors have reported the effects of E-O_3_ on yield, physiological and biochemical responses (particularly photosynthetic characteristics), and reproduction in rice [4,10,12,19], very little research has studied the effect of E-O_3_ on leaf structure. To our knowledge, no data are available on the effects of E-O_3_ on the leaf structure of transgenic rice. Because of the rapid increase in planted areas of transgenic crops worldwide, more attention should be paid to assessing the ecological and environmental risk of transgenic crops under the projected future E-O_3_ climate. Therefore, in this study, we conducted preliminary studies on changes in the leaf surface structure and inner ultrastructure in Bt-SY63 and SY63 under E-O_3_. 

### Surface structure responses of Bt-SY63 and SY63 to elevated O_3_

Our studies found that there were no significant differences in stomatal length, stomatal density and leaf thickness between Bt-SY63 and SY63. However, the leaf area of Bt-SY63 was smaller than that of SY63 owing to smaller leaf length (no significant difference in leaf width between Bt-SY63 and SY63). Shu et al.[22] and Kim et al.[23] reported that the insertion of the cry1Ab and/or cry1Ac genes into the rice genome caused phenotypic variations such as reduced plant height and shorter root length. The results of the present study indicate that the incorporation of cry1Ab/Ac into SY63 can result in unintentional changes in, at least, leaf length and leaf area.

Compared to the A-O_3_ condition, the leaf length, leaf width, leaf area, stomatal length and stomatal density of Bt-SY63 and SY63 were not significantly affected by E-O_3_. However, the significant combined effect of O_3_ and variety on leaf thickness suggested the response of transgenic Bt rice to E-O_3_ with respect to leaf thickness was different from that of its non-transgenic isoline: the leaf thickness of Bt-SY63 increased in response to E-O_3_, while that of SY63 decreased. The leaf thickness of SY63 in response to E-O_3_ is consistent with the response of cotton (*Gossypium hirsutum* L) and birch (*Betula pendula*) under E-O_3_ [24,25], whereas the response of Bt-SY63 observed in this study was opposite to the results of cotton and birch. The response of transgenic Bt rice to E-O_3_ was different from that of conventional rice with respect to leaf thickness, which may be attributed to the incorporation of the transgenes.

### Responses of the leaf ultrastructure of Bt-SY63 and SY63 to elevated O_3_

The cell wall thickness of the mesophyll of Bt-SY63 was smaller than that of SY63, which was probably attributed to the incorporation of the transgenes. E-O_3_ had no significant effect on the cell wall thickness of the two rice genotypes. 

Damaged nuclear envelopes and swollen thylakoids appeared at a later stage (102-DAS) in the two rice genotypes under A-O_3_. Moreover, the chloroplast envelope of Bt-SY63 but not SY63 was injured on 102-DAS, and part of the chloroplasts of the two varieties remained in the grana under A-O_3_.Under E-O_3_, the nuclear membrane of both Bt-SY63 and SY63 became damaged on 85-DAS, swollen thylakoids appeared on 64-DAS for Bt-SY63 and on 85-DAS for SY63 under E-O_3_ instead of on 102-DAS for the two rice genotypes under A-O_3_. Severe damage caused by O_3_ to the chloroplast structure of the two varieties was noted on 102-DAS, concomitant with the disintegration of the grana. Furthermore, the negative effect of O_3_ on chloroplasts was more prominent for Bt-SY63 compared to SY63: mesophyll cells of Bt-SY63 contained completely disintegrated and metamorphous chloroplasts, while the chloroplasts of SY63 still maintained a certain shape. It is well known that the swelling of thylakoids is also a typical feature during leaf senescence. Thus, thylakoid swelling is thought to result from stress-induced senescence [26]. Earlier swelling of thylakoids and damage to the nuclear membrane, accompanied by a breakdown of chloroplast grana, suggest that E-O_3_ accelerated the leaf senescence of the two genotypes rice. The swelling of thylakoids directly resulted in the swelling of chloroplasts, and then resulted in the increase of CCAP (*P* > 0.05) on 64-DAS and subsequently significant increase of CCAP (*P* < 0.01) on 85-DAS in E-O_3_ versus A-O_3_. In fact, the increase of CCAP at these two stages is likely not an example of an increase in active photosynthetic membranes and, instead, may be a result of membrane deterioration. The markedly larger volatility in CCAP of Bt-SY63 (114.5%) compared with SY63 (23.4%) as well as the significant interaction (*P* < 0.01) between O_3_ and variety in CCAP suggested higher O_3_ sensitivity of Bt-SY63. Furthermore, the more severe breakdown of chloroplasts and earlier appearance of swollen thylakoids in Bt-SY63 in comparison to SY63 under E-O_3_ indicated that the injury induced by E-O_3_ for Bt-SY63 was greater than that for SY63. These differences may result for the following reasons: (1) These differences may be related to the amount of mesophyll tissues in the two rice genotypes, such as longer leaf length and larger leaf area in SY63 versus Bt-SY63. A thicker and larger rice leaf that contains more photosynthetic apparatus per unit area [27] would indicate greater photosynthesis potential, which suggests a greater ability to disseminate gases within the leaf as well as a greater potential detoxifying capacity [26]. (2) Bt-SY63 itself undergoes earlier senescence than SY63, which was supported by the onset of earlier heading in Bt-SY63 than in SY63 by 5-7 d under A-O_3_.

Plastoglobuli are thought to be lipid components of membranes that are synthesized but not utilized in thylakoid biosynthesis [28]. It has been proposed that plastoglobuli are composed of the products of membrane breakdown [29]. Significant increases in PCAP both on 64-DAS and on 85-DAS were caused by E-O_3_, suggesting that E-O_3_ accelerated the thylakoid degradation of the two genotypes, Bt-SY63 and SY63 [30]. Moreover, The markedly larger volatility in PCAP of Bt-SY63 (177.9%) compared with SY63 (44.9%) as well as the significant interaction (*P* < 0.05) between O_3_ and variety in PCAP suggested that Bt-SY63 is more sensitive to E-O_3_ than SY63. In addition, altered thylakoid membrane structure may directly affect membrane functionality and could have deleterious effects on the photosynthetic activities of chloroplast [31]. Therefore, in the present experiment, the thylakoid membrane degradation induced by E-O_3_ could lead to deterioration in the photosynthetic activities of the chloroplasts of the two genotypes rice. This supposition is consistent with the data that ‘E-O_3_ led to a significant decline in the Pn of both Bt-SY63 and SY63 in comparison with A-O_3_’ [19], which is in accord with the results of our present study. 

Starch accumulation may indicate an increase in the photosynthetic activity and/or the inhibition of carbohydrate transport out of the leaves to other organs. The decline in the amount of starch grains (SCAP) as the photosynthate for both Bt-SY63 and SY63 under E-O_3_ could be attributable to the decrease in the photosynthetic activities of the chloroplasts of both the genotypes caused by E-O_3_ (from [19]). Moreover, the more prominent decline of SCAP in Bt-SY63 (55.9%) compared with SY63 (31.4%) indicates greater damage to Bt-SY63 than to SY63 under the same O_3_ stress. Moreover, a significant combined effect of O_3_ and variety on SCAP (*P* < 0.05) at 85-DAS indicated that Bt-SY63 was more susceptible to E-O_3_ than SY63. 

## Conclusions

In summary, the results of our study suggest that the incorporation of cry1Ab/Ac into SY63 could induce unintentional changes in some parts of morphology of plant, including leaf length, leaf area and mesophyll cell wall thickness. Furthermore, E-O_3_ induced opposite changes with respect to the leaf thickness of the two genotypes of rice. E-O_3_ had no significant effects on leaf length, leaf width, leaf area, stomatal length, stomatal density and cell wall thickness for both Bt-SY63 and SY63. E-O_3_ caused a significant increase in PCAP and a significant decrease in SCAP accompanied by both swollen chloroplast thylakoids and stroma granulation for the two genotypes, and the grana disappeared during the last growth stage. These results are consistent with previous observations of the effects of O_3_ reported for *Pinus sylvestris* L., *Picea abies* L. and others [16,28,32,33]. However, compared with SY63, E-O_3_ resulted in the early swelling of chloroplast thylakoids, greater breakdown of chloroplasts as well as markedly larger volatility in CCAP, PCAP and SCAP in Bt-SY63 versus in SY63. These results suggest that Bt-SY63 is more sensitive to E-O_3_ than SY63 with the former coupled with greater damage.
